# Nonlinear correlation between fatty liver index and carotid intima media thickness among individuals undergoing health examination

**DOI:** 10.3389/fendo.2023.1120581

**Published:** 2023-03-28

**Authors:** Yuanchen Zhou, Shaojie Duan, Rongrui Wang, Jialiang Chen, Shukun Yao

**Affiliations:** ^1^ Peking University China-Japan Friendship School of Clinical Medicine, Peking University, Beijing, China; ^2^ Department of Gastroenterology, China-Japan Friendship Hospital, Beijing, China; ^3^ Graduate School of Beijing University of Chinese Medicine, Beijing, China; ^4^ Center of Integrative Medicine, Beijing Ditan Hospital, Capital Medical University, Beijing, China

**Keywords:** fatty liver index (FLI), carotid intima-media thickness, J curve, health examination, athrosclerosis

## Abstract

**Background:**

Fatty liver index (FLI) is a predictor of non-alcohol fatty liver disease (NAFLD). This study aimed to assess the association between FLI and carotid intima media thickness (CIMT).

**Methods:**

In this cross-sectional study, we enrolled 277 individuals for health examination from the China-Japan Friendship Hospital. Blood sampling and ultrasound examinations were conducted. Multivariate logistic regression and restricted cubic spline analyses were performed to evaluate the association between FLI and CIMT.

**Results:**

Overall, 175 (63.2%) and 105 (37.9%) individuals had NAFLD and CIMT, respectively. The multivariate logistic regression analyses results showed that high FLI was independently associated with a high risk of increased CIMT, T2 vs. T1 (odds ratio [OR], 95% confidence interval [CI]): 2.41, 1.10–5.25, p = 0.027; T3 vs. T1 (OR, 95% CI): 1.58, 0.68–3.64, p = 0.285. The association between FLI and increased CIMT exhibited a J-shaped curve (nonlinear, p = 0.019). In the threshold analysis, the OR for developing increased CIMT was 1.031 (95% CI: 1.011–1.051, p = 0.0023) in participants with FLI < 64.247.

**Conclusion:**

The relationship between FLI and increased CIMT in the health examination population is J-shaped, with an inflection point of 64.247.

## 1 Introduction

Non-alcoholic fatty liver disease (NAFLD) is characterized by fatty liver that is related to over-nutrition in the absence of excessive alcohol consumption (1, 2). NAFLD is now the most common liver disease with a prevalence of approximately 20–30% among adults worldwide, and a very high disease burden in Asia (3). NAFLD is growing at a rate that is parallel to that of the obesity epidemic, and people with NAFLD have increased risk of cardiovascular disease (CVD) according to meta-analyses of large observational studies (4). Subclinical atherosclerosis, an early stage of CVD, is of vital clinical significance. The onset of clinical CVD events can be delayed or prevented if screening and precautionary measures are available at this stage (5). Accordingly, metrics that indicate poor cardiovascular prognosis, such as increased carotid intima media thickness (CIMT) for arterial wall thickness (6) and increased brachial-ankle pulse wave velocity for stiffness (7), are particularly important in patients with NAFLD.

NAFLD is diagnosed using modalities, such as ultrasound, computed tomography (CT), magnetic resonance imaging (MRI), and liver biopsy. Non-invasive serum-based methods of diagnosing hepatic steatosis, such as fatty liver index (2, 8) (FLI, an index based on body mass index [BMI]), waist circumference, triglyceride level, and gamma glutamyl transferase (GGT) level, were mentioned in previous studies, although their diagnostic cut-off values were not consistent. FLI might serve as a prognostic indicator of death and morbidity including CVD, cancer, respiratory disease, and liver disease (9). Hepatic steatosis index and FLI are independently correlated with carotid atherosclerosis in patients with type 2 diabetes mellitus (10). However, whether FLI influences the risk of increased carotid intima-media thickness (CIMT) during health examination remains unknown.

## 2 Methods and materials

### 2.1 Data sources and study population

**Figure 1:**
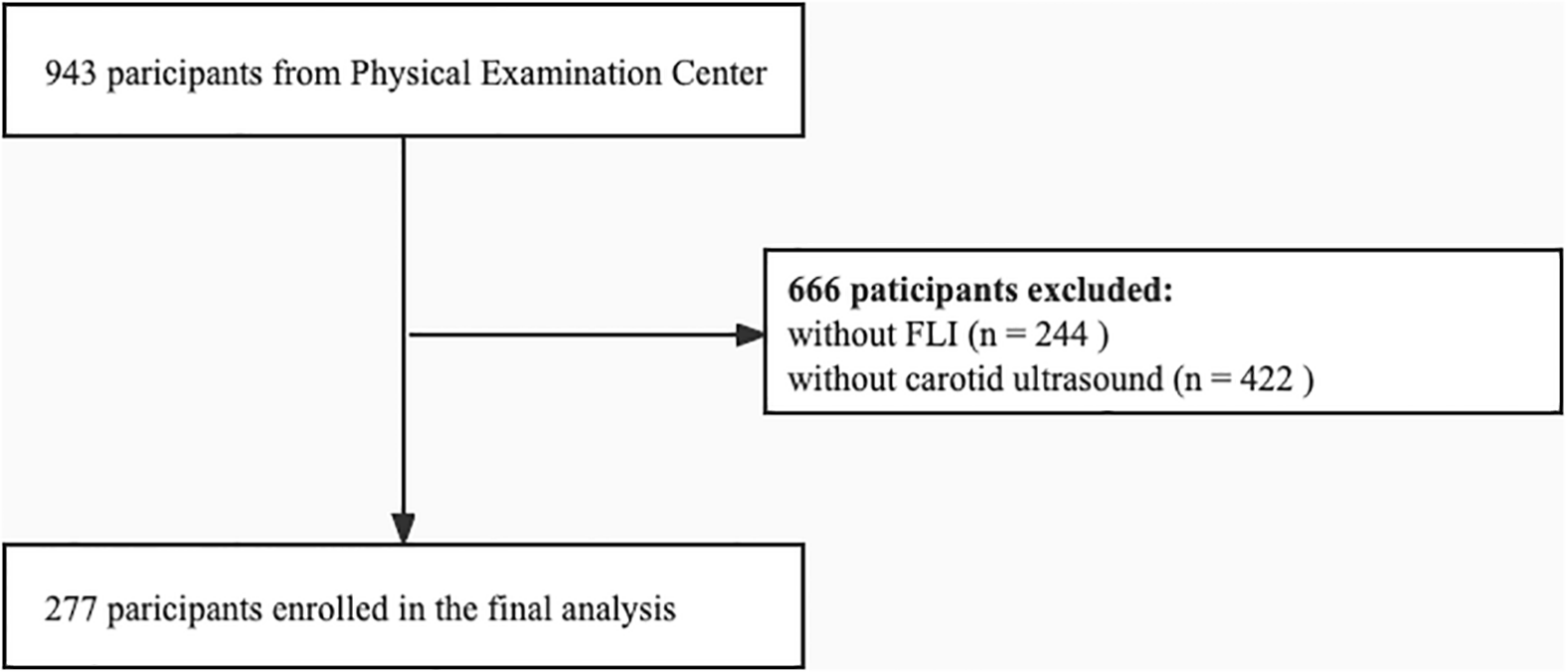
Flowchart of patient selection. FLI, fatty liver index.

This cross-sectional study was conducted at the Health Examination Center of China-Japan Friendship Hospital in Beijing, China. We continuously recruited 943 individuals who underwent health examinations, including anthropometry, laboratory tests, and liver and carotid ultrasound examination, between September 2018 and October 2021. Standardized questionnaires (to collect data, such as demographic information, lifestyle, and history of disease) were completed under the guidance of the researchers. We determined whether a participant had increased CIMT based on the reports of carotid ultrasound examination, and data on FLI was collated. Six hundred and sixty-six participants without data on FLI and carotid ultrasound examination were excluded. Pregnant and lactating women; participants with a history of severe brain disease, coronary heart disease, lung disease, kidney disease, blood disease, psychiatric disease, infectious disease, and malignancy; and those with missing information were excluded. Finally, 277 participants who underwent carotid ultrasound were recruited (**Figure 1**). This study was conducted in accordance with the Declaration of Helsinki and was approved by the Clinical Research Ethics Committee of China-Japan Friendship Hospital (2018-110-K79-1). All participants voluntarily agreed to participate in this study and provided written informed consent.

### 2.2 Anthropometric and biochemical measurements

The health examinations were performed in the morning. Anthropometric indicators were measured by professionally trained physicians. Height, weight, and waist circumference were measured while the participants were in the standing position without shoes and heavy clothing. After 10 min of rest, the blood pressure was measured using an upper arm electronic sphygmomanometer. Fasting blood samples were stored and measured in the laboratory of China-Japan Friendship Hospital. Laboratory investigation data were obtained from the electronic medical record system, including data on total cholesterol, triglyceride, high-density lipoprotein cholesterol (HDL-C), low-density lipoprotein cholesterol (LDL-C), fasting blood glucose (FBG), glycosylated hemoglobin (HbA1c), alanine aminotransferase, aspartate aminotransferase, GGT, and alkaline phosphatase.

The determination of previous disease (hypertension, NAFLD, and diabetes) was based on a question in the questionnaire that asked whether they had been previously informed by a doctor that they had the condition. Body mass index (BMI) was calculated as the weight (in kilograms) divided by the square of the height (in meters). Waist circumference was the circumference at the level of the flat navel. FLI was calculated using a previously published formula (8).

### 2.3 Determination of CIMT and carotid plaque

Carotid atherosclerosis was evaluated using high-resolution ultrasonography to determine the atherosclerotic indices of intima media thickness in the common carotid arteries, carotid artery bulbs, and internal and external carotid arteries. CIMT was measured from the upper edge of the clavicle to the lower edge of the mandible. The mean-intima media thickness was defined as the mean intima media thickness of the proximal and distal walls of both common carotid arteries on a longitudinal scan at a point 10 mm proximal to the beginning of the dilation of each carotid artery bulb. A single trained medical technologist performed all examinations using high-resolution ultrasonography with a 7.5-MHz transducer that produced an axial resolution of 0.1 mm (Siemens, Erlangen, Germany). In this study, the average value was considered the final CIMT value. Normal CIMT was defined as CIMT < 1.0 mm, whereas carotid intima-media thickening was defined as CIMT ≥ 1.0 mm as previously described. Based on the results of carotid ultrasonography, participants with carotid plaques were classified as the plaque group and those without carotid plaques were classified as the non-plaque group.

### 2.4 Statistical analyses

Baseline characteristics are described as the mean (standard deviation) and median (interquartile range) for continuous variables, and numbers (percentage) for categorical variables. One-way Analysis of Variance (ANOVA) or Kruskal–Wallis H test was used for continuous variables, and Chi-square or Fisher’s exact test was used for categorical variables to determine differences between the groups. Prior to regression analysis, missing values for covariates were imputed using multiple imputations of the fully conditional normative (FCS-MI) method. This method enables the specification of multivariate imputation models on a variable-by-variable basis and uses a principled but malleable approach to address missing data. Five datasets were established and modeled individually, with missing data imputed.

Univariate and multivariate logistic regression models were used to determine the odds ratios (OR) and 95% confidence intervals (CIs) for the relationship between FLI and CIMT. We selected these confounders based on their association with the outcomes of interest or a change in effect estimate of more than 10% (11). Model 1: adjusted for age and sex; Model 2: adjusted for variables included in Model 1, smoking, drinking, systolic blood pressure, diastolic blood pressure, HDL-C, and HbA1c; and Model 3: adjusted for variables included in Model 2, hypertension history, and NAFLD history. Additionally, restricted cubic spline regression was performed with four knots at the 5th, 35th, 65th, and 95th percentiles of FLI to assess linearity and examine the curve between FLI and CIMT. We used a two-piece-wise logistic regression model with smoothing to analyze the association threshold between FLI and CIMT after adjusting the variables in Model 3. The likelihood-ratio test and the bootstrap resampling method were used to determine the inflection points.

In sensitivity analyses, the analysis was repeated for data without multiple imputation. Furthermore, potential modifications of the relationship between FLI and CIMT were assessed, including the following variables: sex, age (< 45 vs. >50 years), hypertension history (yes or no), and NAFLD history (yes or no). Heterogeneity among subgroups was assessed using multivariate logistic regression analysis, and interactions between subgroups and FLI were examined using likelihood ratio testing. All analyses were performed using the statistical software packages R 3.3.2 (http://www.R-project.org, The R Foundation, Shanghai, China) (accessed on March 10, 2022) and Free Statistics software version 1.7 (12). A descriptive study was conducted on all participants. A two-tailed p-value <0.05 was considered significant.

## 3 Results

### 3.1 Demographics and baseline information

In total, 277 individuals had sufficiently reliable data to meet the inclusion criteria of our study. **Table 1** illustrates the baseline characteristics of all subjects according to their FLI. There were 105 (37.9%) individuals with carotid intima media thickness. The average age of the study participants was 47.1 (10.9) years, and 212 (76.5%) were male. Higher FLI was associated with increased smoking and higher systolic blood pressure, diastolic blood pressure, FBG, HbA1c, alanine aminotransferase, aspartate aminotransferase, and alkaline phosphatase levels (P < 0.001 for all).

**Table 1:** Baseline characteristics of the study participants stratified by the tertiles of the FLI.

Characteristic	FLI				
	Total	T1 (≤38.70)	T2 (38.70-67.45)	T3 (≥67.45)	*P*-value
No.	277	92	92	93	
Age, years	47.1 ± 10.9	45.3 ± 11.5	47.9 ± 11.1	48.2 ± 10.0	0.145
Sex, Male (%)	212 (76.5)	59 (64.1)	73 (79.3)	80 (86)	0.002
Smoking, n (%)	99 (35.7)	17 (18.5)	37 (40.2)	45 (48.4)	< 0.001
Drinking, n (%)	93 (33.6)	26 (28.3)	30 (32.6)	37 (39.8)	0.245
Hypertension History, n (%)	177 (63.9)	44 (47.8)	57 (62)	76 (81.7)	< 0.001
NAFLD History, n (%)	161 (58.1)	43 (46.7)	58 (63)	60 (64.5)	0.025
DM, n (%)	9 (3.2)	4 (4.3)	2 (2.2)	3 (3.2)	0.764
NAFLD, n (%)	175 (63.2)	30 (32.6)	67 (72.8)	78 (83.9)	< 0.001
Pulse (bpm)	75.5 ± 10.5	72.7 ± 10.2	77.4 ± 10.5	76.5 ± 10.3	0.006
SBP (mmHg)	132.9 ± 16.8	126.7 ± 16.5	132.9 ± 15.3	138.9 ± 16.4	< 0.001
DBP (mmHg)	81.7 ± 12.4	75.9 ± 12.7	81.6 ± 9.8	87.6 ± 11.8	< 0.001
WC (cm)	93.7 ± 9.5	85.2 ± 7.2	93.7 ± 5.9	102.0 ± 6.6	< 0.001
HC (cm)	103.2 ± 6.7	98.6 ± 5.7	103.9 ± 5.0	107.0 ± 6.4	< 0.001
BMI (Kg/m^2^)	27.0 ± 3.2	24.3 ± 2.3	26.9 ± 1.7	29.7 ± 3.0	< 0.001
TC (mmol/L)	4.8 (4.2, 5.5)	4.8 (4.3, 5.5)	4.6 (4.2, 5.5)	4.8 (4.2, 5.4)	0.812
TG (mmol/L)	1.6 (1.1, 2.2)	1.0 (0.7, 1.3)	1.6 (1.3, 2.0)	2.4 (1.8, 3.3)	< 0.001
HDL-C (mmol/L)	1.2 (1.1, 1.4)	1.4 (1.2, 1.6)	1.2 (1.0, 1.4)	1.1 (1.0, 1.3)	< 0.001
LDL-C (mmol/L)	2.8 (2.4, 3.4)	2.8 (2.4, 3.4)	2.8 (2.4, 3.6)	2.8 (2.4, 3.4)	0.433
FBG (mmol/L)	5.8 ± 1.5	5.4 ± 1.0	5.7 ± 1.3	6.4 ± 1.9	< 0.001
HbA1c (%)	5.7 ± 0.8	5.6 ± 0.6	5.6 ± 0.6	5.9 ± 1.0	< 0.001
Antihypertension Drugs, n (%)	17 (6.1)	5 (5.4)	4 (4.3)	8 (8.6)	0.456
ALT (IU/L)	29.0 (21.0, 40.0)	22.0 (16.8, 28.2)	30.0 (21.0, 39.2)	37.0 (26.0, 52.0)	< 0.001
AST, (IU/L)	22.0 (18.0, 27.0)	20.0 (17.8, 23.0)	21.0 (19.0, 26.0)	25.0 (21.0, 30.0)	< 0.001
TBil, (μmol/L)	12.4 (9.4, 16.3)	12.6 (9.5, 16.6)	12.3 (8.8, 16.1)	12.6 (9.7, 16.3)	0.724
DBil, (μmol/L)	1.9 (1.6, 2.4)	1.8 (1.5, 2.3)	2.0 (1.5, 2.5)	2.0 (1.6, 2.4)	0.268
ALP, (IU/L)	70.0 (61.0, 82.0)	65.0 (57.0, 79.0)	68.5 (61.8, 81.5)	77.0 (64.0, 86.0)	0.001
GGT, (IU/L)	28.0 (20.0, 42.0)	19.0 (14.0, 25.2)	28.0 (23.0, 34.2)	47.0 (29.0, 73.0)	< 0.001
CIMT, n (%)	105 (37.9)	24 (26.1)	42 (45.7)	39 (41.9)	0.015
Carotid Plaques, n (%)	111 (40.1)	31 (33.7)	42 (45.7)	38 (40.9)	0.25

Data are presented as means ± SD or medians (inter-quantile range (IQR)) for continuous variables and number (percentages) for categorical variables. FLI, fatty liver index; BMI, body mass index; T, tertiles; DM, diabetes mellitus; NAFLD, non-alcoholic fatty liver disease; SBP, systolic blood pressure; DBP, diastolic blood pressure; WC, waist circumference; HC, hip circumference; BMI, body mass index; TC, Total Cholesterol; TG, triglyceride; HDL-C, high-density lipoprotein cholesterol; LDL-C, low-density lipoprotein cholesterol; FBG, fasting blood glucose; HbA1c, glycosylated hemoglobin; ALT, alanine aminotransferase; AST, aspartate aminotransferase; TBil, total bilirubin; DBil, direct bilirubin; GGT, γ-glutamyl transpeptidase; ALP, alkaline phosphatase; CIMT, carotid intima media thickness.

### 3.2 Nonlinear correlation between FLI and CIMT

**Figure 2:**
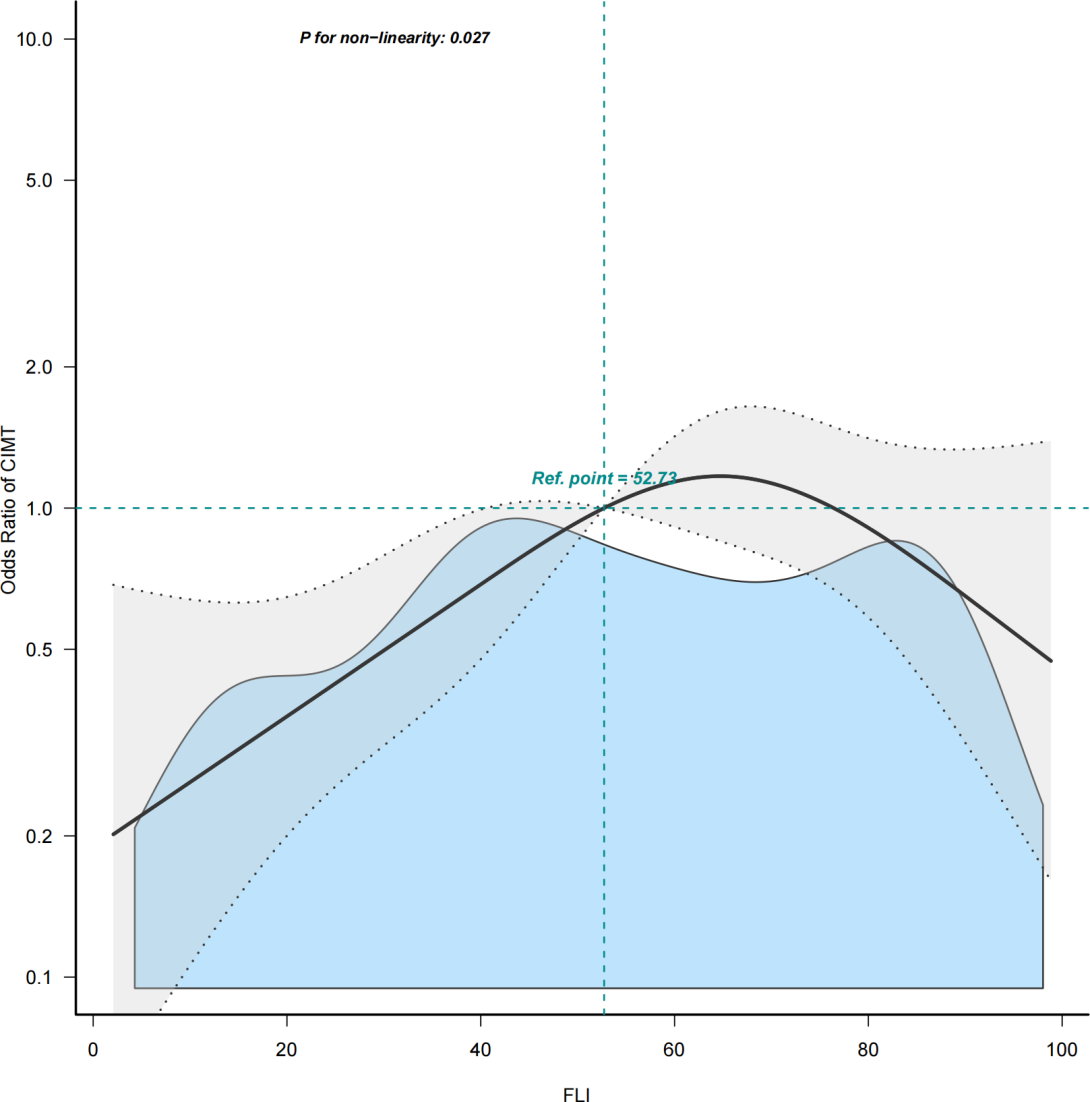
Restricted cubic spline plot of the association between FLI and CIMT. Solid and dashed lines represent the predicted and 95% confidence interval. FLI, fatty liver index; CIMT, carotid intima-media thickness.

The multivariable logistic regression analyses results of the associations between FLI and CIMT is presented in **Table 2**. The analysis was performed after adjusting for confounding factors, including age, sex, smoking, drinking, systolic blood pressure, diastolic blood pressure, HDL-C, HbA1c, hypertension history, and NAFLD history. Compared with individuals with lower FLI T1 (≤ 38.70), the adjusted OR values for FLI and CIMT in T2 (38.70–67.45) and T3 (≥ 67.45) were 2.41 (95% CI: 1.10–5.25, *p* = 0.027), and 1. 58 (95% CI: 0.68–3.64, *p* = 0. 285), respectively. Accordingly, the association between FLI and CIMT exhibited a J-shaped curve (nonlinear, *p* = 0. 027) in the restricted cubic spline (**Figure 2**). In the threshold analysis, the OR of developing CIMT was 1.031 (95% CI: 1.011–1.051, *p* = 0.002) in participants with FLI < 64.247 (**Table 3**). This means that the risk of increased CIMT increases by 3.1% with every 1 unit increase in FLI. The association between FLI and CIMT was stable when the FLI was ≥ 64.247 (**Table 3**).

**Table 2:** Multivariate logistic regression analyses of associations between FLI and CIMT.

	No.	Unadjusted		Model 1		Model2		Model3	
		OR (95%CI)	*P*-value	OR (95%CI)	*P*-value	OR (95%CI)	*P*-value	OR (95%CI)	*P*-value
FLI	277	1.01 (1.00~1.02)	0.0190	1.01 (0.997~1.02)	0.120	1.01 (0.99~1.02)	0.220	1.01 (0.995~1.02)	0.210
T1	92	Ref		Ref		Ref		Ref	
T2	92	2.38 (1.28~4.43)	0.006	2.23 (1.09~4.55)	0.028	2.27 (1.06~4.85)	0.034	2.41 (1.10~5.25)	0.027
T3	93	2.05 (1.1~3.81)	0.024	1.79 (0.87~3.67)	0.111	1.56 (0.68~3.56)	0.296	1.58 (0.68~3.64)	0.285
P for trend			0.027		0.138		0.351		0.357

Model 1: Adjust for age and sex.

Model 2: Adjust for Smoking, Drinking, SBP, DBP, HDL-C, HbA1c in addition to model 1.

Model 3: Adjust for Hypertension history, NAFLD history in addition to model 2.

FLI, fatty liver index; CIMT, carotid intima media thickness; T, tertiles; OR, odds ratio; CI, confidence interval; Ref, reference; SBP, systolic blood pressure; DBP, diastolic blood pressure; HDL-C, high-density lipoprotein cholesterol; HbA1c, glycosylated hemoglobin; NAFLD, non-alcoholic fatty liver disease.

**Table 3:** Threshold effect analysis of the relationship of FLI with CIMT.

FLI	Model	
	OR (95% CI)	*P*-value
<64.247	1.031(1.011 – 1.051)	0.0023
≥64.247	0.966(0.924 – 1.009)	0.1229
log likelihood ratio test		0.008

FLI, fatty liver index; CIMT, carotid intima media thickness; OR, odds ratio; CI, confidence interval.

### 3.3 Stratified and sensitivity analysis

In several subgroups, stratified analysis was performed to assess the potential effect of modifications on the relationship between FLI and CIMT. No significant interactions were found in any subgroups after stratifying by age, sex, NAFLD history, and hypertension history (**Supplementary Figure 1**). In sensitivity analyses, results obtained without using multiple imputations remained consistent (**Supplementary Tables 1, 2**). Compared with individuals with lower FLI in T1 (≤38.70), the adjusted OR values for FLI and CIMT in T2 (38.70–67.45) and T3 (≥67.45) were 3.32 (95% CI: 1.00–11.04, *p* = 0.05), and 1.67 (95% CI: 0.39–7.24, *p* = 0. 491), respectively.

## 4 Discussion

This cross-sectional study of participants who underwent health examination demonstrated a J-shaped relationship between FLI and CIMT, with an inflection point of approximately 64.247. Both the stratified and sensitivity analyses showed that the relationship between FLI and CIMT remained robust.

The leading cause of death in individuals with NAFLD is CVD (13). A recent meta-analysis reported that NAFLD was associated with a moderately increased risk of fatal or non-fatal CVD events (pooled HR: 1.45, 95% CI: 1.31–1.61). This risk markedly increases across the severity of NAFLD, especially in the fibrosis stage (pooled HR: 2.50, 95% CI: 1.68–3.72) (4). The mechanism by which steatosis increases the risk of CVD is unclear. Cardiometabolic disease, characterized by metabolic disorder triggered cardiovascular events, is a leading cause of death and disability. However, CVD events are a late step in the process of atherogenesis which makes it difficult to ascertain the contribution of steatosis itself.

We postulated that if steatosis played an independent role in the development of atherosclerosis, it should promote the occurrence and progression of early, pre-atherosclerotic lesions. In Familial Combined Hyperlipidemia patients with more severe steatosis, the risk of atherosclerotic plaque development was significantly increased in patients with liver fibrosis, suggesting that dyslipidemia and insulin resistance may be processes between liver disease and atherosclerotic damage (14). Metabolic syndrome could worsen CIMT in patients with NAFLD (15). In rats with NASH, CIMT correlated with hepatic inflammation score (16). Moreover, mechanism studies have found that the accumulation of fat in the liver could increase free fatty acid levels, which are involved in inflammatory responses and endothelial injury during the development of atherosclerosis (17). NAFLD might accelerate the progress of increased CIMT by a common metabolic dysfunction, such as insulin resistance, glucose and lipid metabolism disorders (18, 19), and low-grade inflammation (20).

FLI is a predictor of NAFLD and is calculated using serum triglyceride and GGT levels, BMI, and waist circumference. FLI integrates obesity, central obesity, lipid metabolism disorders and GGT, which might serve as a marker of metabolic dysfunction (21) and low-grade inflammation (22–24). Furthermore, non-invasive hepatic steatosis indices are positively correlated with fasting blood insulin, C peptide, triglyceride, total cholesterol, and LDL-C levels, but negatively correlated with HDL-C levels, which are also associated with atherosclerosis. In this study, we demonstrated that a threshold effect was presented by restricted cubic spline, which might be an indicator of CIMT, a pre-atherosclerotic lesion that predicts cardiovascular events. Therefore, we believe that NAFLD may contribute to the occurrence and development of carotid atherosclerosis by aggravating metabolic dysfunction and inflammation.

Predicting early atherosclerosis plays an important role in decreasing CVD events. A J-shaped relationship was demonstrated between FLI and CIMT in this study. The CIMT increased proportionally with FLI, and this association was independent of traditional cardiometabolic risk factors.

Our study has several limitations. First, this was a cross-sectional study and the results of this study should not be used to draw causal conclusions. Second, the overall sample size of our study was small due to missing data from laboratory results. Third, even though regression models were constructed, and stratified analyses and sensitivity analysis were performed, residual confounding effects from unmeasured or unknown factors could not be entirely excluded. Therefore, further studies between FLI and CIMT are required in the future.

In conclusion, the results of this study indicate that FLI, which is readily available in clinical practice, may be more useful as a risk assessment index for CIMT among people undergoing health examination. This may have clinical utility and guide treatment choices.

## Data availability statement

The original contributions presented in the study are included in the article/**Supplementary Material**. Further inquiries can be directed to the corresponding authors.

## Ethics statement

The studies involving human participants were reviewed and approved by clinical research ethics committee of China-Japan Friendship Hospital. The patients/participants provided their written informed consent to participate in this study.

## Author contributions

YZ, data curation, software, writing - review and editing. SD and RW, data curation, writing - review and editing. JC and SY, conceptualization, methodology, investigation, resources, data curation, visualization, supervision, funding acquisition, writing - review and editing.

## References

[1] Murag S Ahmed A Kim D. Recent epidemiology of nonalcoholic fatty liver disease. Gut Liver (2021) 15(2):206–16. doi: 10.5009/gnl20127 PMC7960978 32921636

[2] Wong VW Chan WK Chitturi S Chawla Y Dan YY Duseja A. Asia-Pacific working party on non-alcoholic fatty liver disease guidelines 2017-part 1: Definition, risk factors and assessment. J Gastroenterol Hepatol (2018) 33(1):70–85. doi: 10.1111/jgh.13857 28670712

[3] Henry L Paik J Younossi ZM. Review article: The epidemiologic burden of non-alcoholic fatty liver disease across the world. Aliment Pharmacol Ther (2022) 56(6):942–56. doi: 10.1111/apt.17158 35880713

[4] Mantovani A Csermely A Petracca G Beatrice G Corey KE Simon TG. Non-alcoholic fatty liver disease and risk of fatal and non-fatal cardiovascular events: An updated systematic review and meta-analysis. Lancet Gastroenterol Hepatol (2021) 6(11):903–13. doi: 10.1016/S2468-1253(21)00308-3 34555346

[5] Fuster V Lois F Franco M. Early identification of atherosclerotic disease by noninvasive imaging. Nat Rev Cardiol (2010) 7(6):327–33. doi: 10.1038/nrcardio.2010.54 20440291

[6] Gill C Vatcheva KP Pan JJ Smulevitz B McPherson DD Fallon M. Frequency of nonalcoholic fatty liver disease and subclinical atherosclerosis among young Mexican americans. Am J Cardiol (2017) 119(11):1717–22. doi: 10.1016/j.amjcard.2017.03.010 PMC6132248 28395890

[7] Huang RC Beilin LJ Ayonrinde O Mori TA Olynyk JK Burrows S. Importance of cardiometabolic risk factors in the association between nonalcoholic fatty liver disease and arterial stiffness in adolescents. Hepatology (2013) 58(4):1306–14. doi: 10.1002/hep.26495 23703776

[8] Bedogni G Bellentani S Miglioli L Masutti F Passalacqua M Castiglione A. The fatty liver index: A simple and accurate predictor of hepatic steatosis in the general population. BMC Gastroenterol (2006) 6:33. doi: 10.1186/1471-230X-6-33 17081293 PMC1636651

[9] Chung GE Jeong SM Cho EJ Yoo JJ Cho Y Lee KN. Association of fatty liver index with all-cause and disease-specific mortality: A nationwide cohort study. Metabolism (2022) 133:155222. doi: 10.1016/j.metabol.2022.155222 35636583

[10] Wang C Cai Z Deng X Li H Zhao Z Guo C. Association of hepatic steatosis index and fatty liver index with carotid atherosclerosis in type 2 diabetes. Int J Med Sci (2021) 18(14):3280–89. doi: 10.7150/ijms.62010 PMC8364463 34400897

[11] Qu X Yang H Yu Z Jia B Qiao H Zheng Y. Serum zinc levels and multiple health outcomes: Implications for zinc-based biomaterials. Bioact Mater (2020) 5(2):410–22. doi: 10.1016/j.bioactmat.2020.03.006 PMC7114479 32258830

[12] Yang Q Zheng J Chen W Chen X Wen D Chen W. Association between preadmission metformin use and outcomes in intensive care unit patients with sepsis and type 2 diabetes: A cohort study. Front Med (Lausanne) (2021) 8:640785. doi: 10.3389/fmed.2021.640785 33855034 PMC8039324

[13] Konyn P Ahmed A Kim D. Causes and risk profiles of mortality among individuals with nonalcoholic fatty liver. Clin Mol Hepatol (2022) 29(Suppl):S43–57. doi: 10.3350/cmh.2022.0351 PMC10029952 36417893

[14] Mandraffino G Morace C Franzè MS Nassisi V Sinicropi D Cinquegrani M. Fatty liver as potential biomarker of atherosclerotic damage in familial combined hyperlipidemia. Biomedicines (2022) 10(8):1770. doi: 10.3390/biomedicines10081770 35892670 PMC9332610

[15] Salvi P Ruffini R Agnoletti D Magnani E Pagliarani G Comandini G. Increased arterial stiffness in nonalcoholic fatty liver disease: The cardio-goose study. J Hypertens (2010) 28(8):1699–707. doi: 10.1097/HJH.0b013e32833a7de6 20467324

[16] Wu J Zhang H Zheng H Jiang Y. Hepatic inflammation scores correlate with common carotid intima-media thickness in rats with nafld induced by a high-fat diet. BMC Vet Res (2014) 10:162. doi: 10.1186/1746-6148-10-162 25030203 PMC4223401

[17] Abdallah LR de Matos RC Souza YPDME Vieira-Soares D Muller-Machado G Pollo-Flores P. Non-alcoholic fatty liver disease and its links with inflammation and atherosclerosis. Curr Atheroscler Rep (2020) 22(1):7. doi: 10.1007/s11883-020-0820-8 32020371

[18] Gaudio E Nobili V Franchitto A Onori P Carpino G. Nonalcoholic fatty liver disease and atherosclerosis. Intern Emerg Med (2012) 7 Suppl 3:S297–305. doi: 10.1007/s11739-012-0826-5 23073871

[19] Katakami N. Mechanism of development of atherosclerosis and cardiovascular disease in diabetes mellitus. J Atheroscler Thromb (2018) 25(1):27–39. doi: 10.5551/jat.RV17014 28966336 PMC5770221

[20] Brouwers M Simons N Stehouwer CDA Isaacs A. Non-alcoholic fatty liver disease and cardiovascular disease: Assessing the evidence for causality. Diabetologia (2020) 63(2):253–60. doi: 10.1007/s00125-019-05024-3 PMC6946734 31713012

[21] Sung KC Ryu S Kim BS Cheong ES Park DI Kim BI. Gamma-glutamyl transferase is associated with mortality outcomes independently of fatty liver. Clin Chem (2015) 61(9):1173–81. doi: 10.1373/clinchem.2015.240424 26152752

[22] Teijeiro A Garrido A Ferre A Perna C Djouder N. Inhibition of the il-17a axis in adipocytes suppresses diet-induced obesity and metabolic disorders in mice. Nat Metab (2021) 3(4):496–512. doi: 10.1038/s42255-021-00371-1 33859430

[23] Wu CK Yang CY Lin JW Hsieh HJ Chiu FC Chen JJ. The relationship among central obesity, systemic inflammation, and left ventricular diastolic dysfunction as determined by structural equation modeling. Obes (Silver Spring) (2012) 20(4):730–7. doi: 10.1038/oby.2011.30 21394094

[24] Soldavini CM Piuri G Rossi G Corsetto PA Benzoni L Maggi V. Maternal Aa/Epa ratio and triglycerides as potential biomarkers of patients at major risk for pharmacological therapy in gestational diabetes. Nutrients (2022) 14(12):2502. doi: 10.3390/nu14122502 35745231 PMC9231064

