# Supplementary material for: Nonlinear correlation between fatty liver index and carotid intima media thickness among individuals undergoing health examination
Source: Front Endocrinol (Lausanne). 2023 Mar 28;14:1120581. doi: 10.3389/fendo.2023.1120581 (PMC10086365; doi:10.3389/fendo.2023.1120581)
Supplement: Supplementary file 1 [file DataSheet_1.docx]

Supplementary Material

Nonlinear Correlation Between Fatty Liver Index and Carotid Intima Media Thickness among Individuals Undergoing Health Examination

Yuanchen Zhou, Shaojie Duan, Rongrui Wang, Jialiang Chen^*^, Shukun Yao^*^

*** Correspondence:** Shukun Yao: shukunyao@126.com, Jialiang Chen: chen0901133@163.com

# Supplementary Figures and Tables

## Supplementary Figures


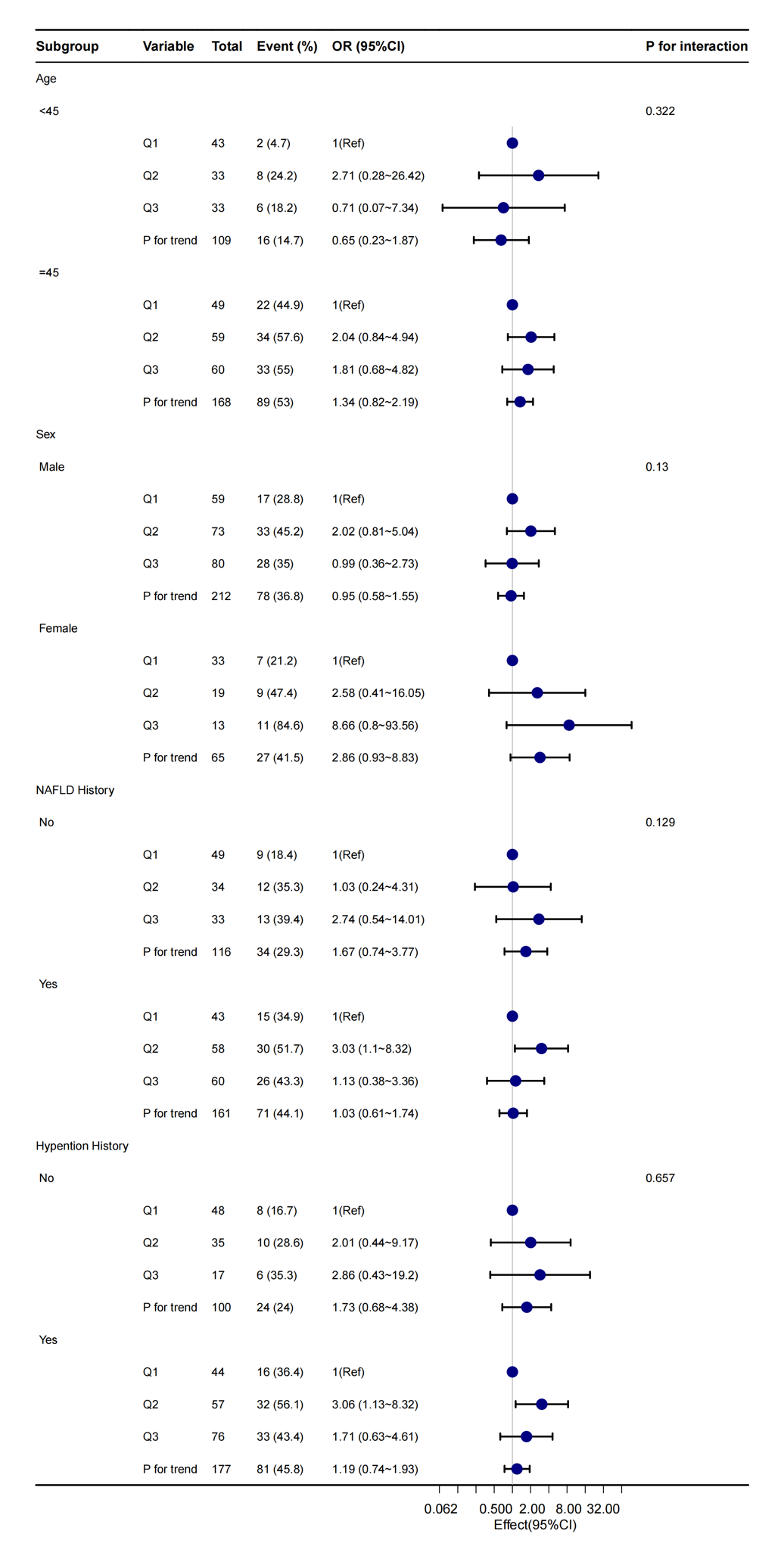


**Supplementary Figure 1.** The relationship between FLI and CIMT according to basic features. Except for stratification component itself, each stratification factor was adjusted for all other variables (Age, Sex, NAFLD History, Hypertension History).

## Supplementary Tables

**Supplementary Table 1**. Univariate of Carotid intima media thickening

| Variable | OR_95CI | P_value |
| --- | --- | --- |
| Age | 1.12 (1.08~1.15) | <0.001 |
| Gender |  |  |
| Male | Reference |  |
| Female | 1.22 (0.69~2.15) | 0.491 |
| Smoking |  |  |
| No | Reference |  |
| Yes | 1.18 (0.71~1.95) | 0.523 |
| Drinking |  |  |
| No | Reference |  |
| Yes | 0.8 (0.47~1.34) | 0.394 |
| History of Hypertension |  |  |
| No | Reference |  |
| Yes | 2.67 (1.55~4.61) | <0.001 |
| NAFLD |  |  |
| No | Reference |  |
| Yes | 1.57 (0.94~2.62) | 0.088 |
| Pulse | 0.99 (0.97~1.01) | 0.449 |
| SBP | 1.02 (1.01~1.04) | 0.003 |
| DBP | 1.02 (1~1.04) | 0.093 |
| Waist Circumference | 1.03 (1.01~1.06) | 0.015 |
| Hip circumference cm | 1.01 (0.97~1.04) | 0.702 |
| BMI | 1.07 (0.99~1.16) | 0.079 |
| TC | 1.2 (0.93~1.54) | 0.153 |
| TG | 1 (0.82~1.21) | 0.991 |
| HDL-C | 1.74 (0.73~4.13) | 0.209 |
| LDL-C | 1.27 (0.94~1.71) | 0.115 |
| FBG | 1.28 (1.06~1.54) | 0.011 |
| ALT | 1 (0.99~1.02) | 0.491 |
| AST | 1.01 (0.99~1.03) | 0.368 |
| TBil | 0.99 (0.95~1.04) | 0.789 |
| DBil | 0.81 (0.56~1.17) | 0.265 |
| ALP | 1.02 (1~1.03) | 0.017 |
| GGT | 1 (1~1.01) | 0.181 |
| HbA1c | 1.83 (1.27~2.63) | 0.001 |
| DM |  |  |
| No | Reference |  |
| Yes | 0.81 (0.2~3.33) | 0.774 |
| Antihypertension Drugs |  |  |
| No | Reference |  |
| Yes | 0.67 (0.23~1.95) | 0.459 |
| FLI |  |  |
| Q1 | Reference |  |
| Q2 | 2.38 (1.28~4.43) | 0.006 |
| Q3 | 2.05 (1.1~3.81) | 0.024 |

Abbreviations: NAFLD, non-alcoholic fatty liver disease; SBP, systolic blood pressure; DBP, diastolic blood pressure; WC, waist circumference; HC, hip circumference; BMI, body mass index; TC, Total Cholesterol; TG, triglyceride; HDL-C, high-density lipoprotein cholesterol; LDL-C, low-density lipoprotein cholesterol; FBG, fasting blood glucose; HbA1c, glycosylated hemoglobin; ALT, alanine aminotransferase; AST, aspartate aminotransferase; TBil, total bilirubin; DBil, direct bilirubin; GGT, γ-glutamyl transpeptidase; ALP, alkaline phosphatase; Q, quartiles.

**Supplementary Table 2**. Multivariate logistic regression analyses of associations between FLI and CIMT.

| Variable | Unadjusted | |  | Model 1 | |  | | Model2 | |  | Model3 | |
| --- | --- | --- | --- | --- | --- | --- | --- | --- | --- | --- | --- | --- |
|  | OR_95CI | P_value |  | OR_95CI | P_value |  | OR_95CI | | P_value |  | OR_95CI | P_value |
| FLI | 1.01 (1.00~1.02) | 0.019 |  | 1.01 (0.9977~1.02) | 0.120 |  | 1.01 (0.99~1.03) | | 0.477 |  | 1.01 (0.99~1.03) | 0.290 |
| Q1 | Ref |  |  | Ref |  |  | Ref | |  |  | Ref |  |
| Q2 | 3.32 (1.67~6.47) | 0.001 |  | 2.89 (1.30~6.42) | 0.009 |  | 2.7 (0.90~8。09) | | 0.077 |  | 3.32 (1.00~11.04) | 0.05 |
| Q3 | 2.69 (1.31~5.50) | 0.007 |  | 2.30 (1.01~5.26) | 0.111 |  | 1.30 (0.33.~5.23) | | 0.707 |  | 1.67 (0.39~7.24) | 0.491 |
| P for trend |  | 0.017 |  |  | 0.114 |  |  | | 0.707 |  |  | 0.663 |

Model 1: Adjust for age and sex.

Model 2: Adjust for Smoking, Drinking, SBP, DBP, TC, HDL-C, LDL-C, FBG, HbA1c, DM in addition to model 1.

Model III: Adjust for Hypertension history, NAFLD history in addition to model 2.

Abbreviations: FLI, fatty liver index; CIMT, carotid intima media thickness; Q, quartiles; OR, odds ratio; CI, confidence interval; Ref, reference; SBP, systolic blood pressure; DBP, diastolic blood pressure; TC, Total Cholesterol; HDL-C, high-density lipoprotein cholesterol; LDL-C, low-density lipoprotein cholesterol; FBG, fasting blood glucose; DM, diabetes mellitus; NAFLD, non-alcoholic fatty liver disease.
